# Desmoplastic small round cell tumors of the pleura: a review of the clinical literature

**DOI:** 10.1186/s40248-017-0103-6

**Published:** 2017-09-09

**Authors:** Alessandro Giuseppe Fois, Pietro Pirina, Antonella Arcadu, Francesca Becciu, Sandra Manca, Viviana Marras, Sara Canu, Gaetano Castagna, Giorgio Carlo Ginesu, Angelo Zinellu, Panagiotis Paliogiannis

**Affiliations:** 1 Department of Clinical and Experimental Medicine, University of Sassari, Viale San Pietro 43, 07100 Sassari, Italy; 2 Department of Biomedical Sciences, University of Sassari, Viale San Pietro 43, 07100 Sassari, Italy

**Keywords:** Pleura, Cancer, Desmoplastic small round cell tumor, DSRCT, EWS-WT1

## Abstract

Desmoplastic small round cell tumor of the pleura is a rare malignancy, with only a few cases reported in the scientific literature. The aim of the present review is to discuss the demographic, pathological, clinical, and therapeutic features of this rare tumor. English-language articles published since 1989, when the first case of desmoplastic small round cell tumor of the pleura was described, were retrieved, and fifteen cases included in fourteen articles were revised. The mean age of the patients was 25.5 years, out of them 60% were males. Chest pain, pleural effusion, and dyspnea were the most common clinical manifestations, while chest roentgenogram and computed tomography were the imaging techniques most commonly used. Surgical biopsy was employed in 80% of the cases for diagnosis. A multidisciplinary approach consisting in a combination of surgery with chemotherapy and radiation therapy was adopted in most cases. Only two patients (13.3%) were alive at 3 years from diagnosis, reflecting the aggressiveness of the disease, and the poor outcomes of the treatments currently available. Desmoplastic small round cell tumors of the pleura are extremely aggressive and challenging to diagnose, because of their rarity and unspecific demographic, clinical, and radiological features. An in-depth knowledge of such features is necessary for the optimal management of patients with this rare malignancy.

## Background

Primary tumors of the pleura are relatively uncommon. The most frequent malignancy is mesothelioma, which is related to asbestos exposure, arises in mesothelial cells, and has a poor prognosis despite recent advances in its multimodality treatment [1, 2]. Desmoplastic small round cell tumor (DSRCT) was first described in 1989 by Gerald and Rosai [3], and Ordonez et al. [4] as a highly aggressive and rare mesenchymal tumor of the serosal surfaces. The main histological features include tightly cohesive nests of small round poorly differentiated malignant cells, with accompanying desmoplastic stromal cells. Chromosomal translocation t (11;22) (p13;q12) is distinctive. Mostly it occurs in the abdomen and pelvis, predominantly in young males [3, 4]. Further anatomical locations, including the pleura, have been reported [5]. The aim of the present review is to describe and discuss the demographic, pathological, clinical, and therapeutic features of the DSRCT of the pleura, as depicted in the current scientific literature.

## Materials and methods

English-language articles published from 1989 to October 2016, and related to pleural round small cell tumor cases were non-systematically retrieved using the Pubmed – Medline database and the Google Scholar general database. The search terms employed were “desmoplastic small round cell tumors”, “pleural desmoplastic round cell tumors”, and “desmoplastic small round cell tumors of the pleura”. Titles and abstracts were evaluated in order to include the most relevant studies. References of the selected articles were cross-checked in order to detect papers missed by the search engine.

## Results

Fifteen cases included in fourteen articles were retrieved [6–19]. Cases with poor clinical data were excluded, as well as those in which the pleural origin was not clearly stated or demonstrated, and those reported in more than one article [6, 8, 20–23]. Additionally, cases included in large series with no detailed information for every specific case were excluded, as were lung cases, even when pleural effusion occurred [24, 25].

The mean age in the whole cohort was 25.5 (range 7–72) years, and nine patients were males (60%, Table 1). Information about the personal clinical history was available in nine cases; one out of them (16.7%) had had a previous trauma, while in the remaining cases no remarkable pathological conditions were found. Data about asbestos exposure and tobacco smoking were available in four cases; two patients were smokers (10- and 7- pack/years history, respectively), and only one of them had a sporadic previous exposure to asbestos.

**Table 1:** Demographic characteristics and clinical manifestations observed in the reviewed cases

Article	N° of cases	Sex	Age	Side	Clinical manifestations
Bian et al., 1993 [6]	1	M	29	Bil	Chest pain, dyspnea, right pleural effusion
Choi et al., 1995 [7]	1	M	33	R	Chest, arm and neck pain, pleural effusion
Parkash et al., 1995 [8]	2	M, F	24, 17	Both L	Chest pain, dyspnea, and pleural effusion in both cases
Venkateswaran et al.,1997 [9]	1	M	16	R	Chest pain, dyspnea, cough, weight loss, effusion
Sàpi et al., 1999 [10]	1	M	25	L	Chest pain, dyspnea
Ostoros et al., 2002 [11]	1	F	19	L	Back pain
Cranja et al., 2005 [12]	1	M	7	NA	Back pain, fever, effusion.
Karavitakis et al., 2007 [13]	1	M	10	L	Back pain
Quarssani et al., 2011 [14]	1	F	19	R	Back pain, cachexia
Benbrahim et al., 2012 [15]	1	F	50	Bil	Cough, dyspnea, effusion
Jian et al., 2014 [16]	1	F	15	R	Chest pain, fever, dyspnea, weight loss, effusion
Cao et al., 2015 [17]	1	F	72	L	Chest pain, dyspnea
Won et al., 2015 [18]	1	M	15	Bil	Chest pain
Ikeue et al., 2016 [19]	1	M	32	L	Cough, chest compression, pleural effusion

*M* males; *F* females; *L* left; *R* right; *Bil* bilateral; *NA* not available

In seven cases (46.7%) the lesions involved the left pleura, in four (26.7%) the right pleura, while in other three cases (20%) the lesions were bilateral (Table 1); in one patient the side of the lesion was not mentioned, but only a mediastinal involvement [12]. In seven cases (70%) the lung was unaffected; as opposed, in five (33.3%) and six (40%) cases respectively pulmonary and mediastinal involvement was seen. Five (33.3%) tumors were located in a paravertebral position, invading the adjacent vertebral bodies. Chest pain (60%), pleural effusion (60%), and dyspnea (46.6%) were the clinical manifestations most frequently encountered (Table 1). Back pain was the main extrathoracic clinical finding, caused probably by the involvement of the thoracic vertebrae.

Data about the radiological evaluation of the cases were available in eleven cases. The imaging techniques most frequently employed were roentgenograms and computed tomography (CT scans), which were used combined in eight cases and alone in three cases each. Magnetic resonance (MRI) and bone scans were used in cases of suspect vertebral involvement. The diagnosis was obtained by surgical biopsy in twelve cases (80%), and needle biopsy in three cases (20%). In two patients, it was evidenced that DSRCT can be diagnosed, or at least suspected, also in the pleural fluid [6, 7]; while in another case it was inconclusive, despite neoplastic cells were detected in the pleural fluid.

**Fig. 1:**
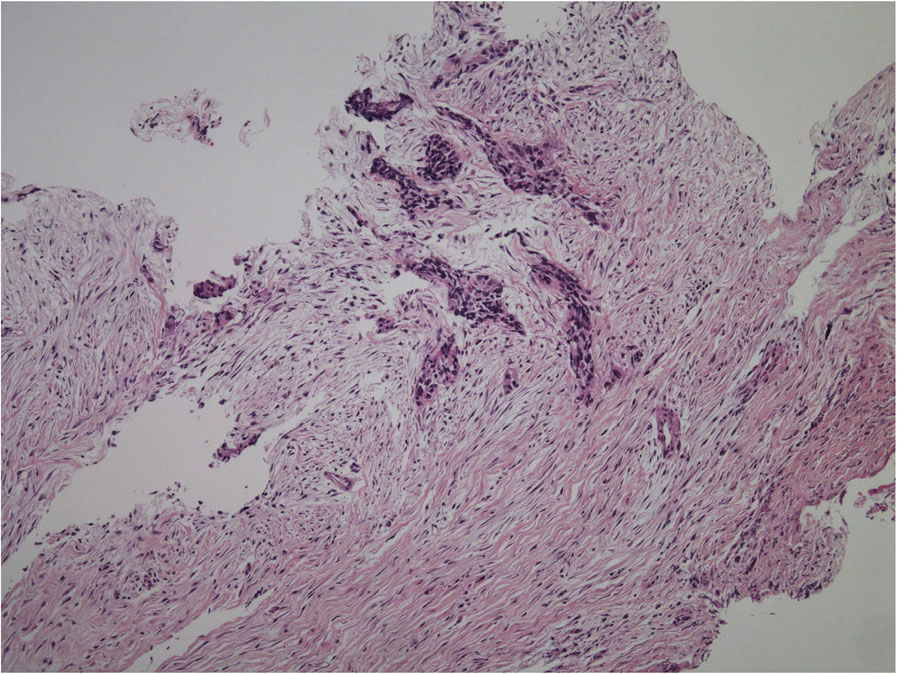
Hematoxylin and Eosin section of a desmoplastic small round cell tumor of the pleura (magnification 4×). (The image was provided by Dr. Tatsuyoshi Ikeue, Department of Respiratory Medicine, Japanese Red Cross Wakayama Medical Center, Wakayama, Japan)

**Fig. 2:**
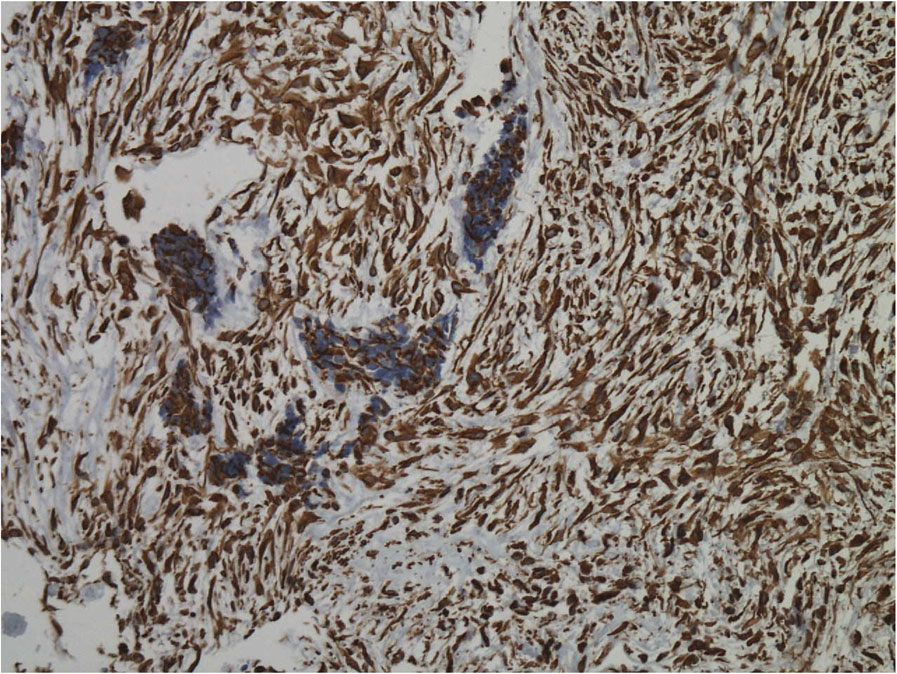
Immunohistochemical positivity for Vimentin in a desmoplastic small round cell tumor of the pleura. (The image was provided by Dr. Tatsuyoshi Ikeue, Department of Respiratory Medicine, Japanese Red Cross Wakayama Medical Center, Wakayama, Japan)

On pathologic examination, the lesions were commonly composed by nests of poorly differentiated closely packed neoplastic cells with small, round to oval hyperchromatic nuclei, and scanty cytoplasms surrounded by an abundant desmoplastic stroma (Fig. 1). This was often rich in vessels, with classic partial thickening of the vascular wall. The mitotic activity was variable, from absent to highly represented (20–25/10 high power field). Unusual features were some abortive glandular structures, the rosette formation, and the papillary structures [9, 11]. Regarding immunohistochemistry, positivity for vimentin was found in all the eleven cases reporting such information (Fig. 2), and positivity for CD99 in four reported cases. Neuron-specific enolase (NSE) and desmin were the immunostainings most frequently used (14 and 13 cases, respectively); the former was positive in 71%, and the latter in 77% of the examined cases (Table 2). Frequent immunostaining for synaptophysin and cytokeratin AE1:AE3 was detected, as well as negativity for smooth muscle actin (SMA), carcinoembryonic antigen (CEA), and chromogranin A. A dot-like pattern, positioned in the nucleus adjacent to the cytoplasm, was often described for vimentin and NSE staining. Desmin immunostaining was variable, ranging from diffuse to focal, and sometimes absent.

**Table 2:** Main immunohistochemistry results in the cohort under investigation

Marker	Positive	Focally positive	Negative	Total
Vimentin	11			11
NSE	10	1	3	14
Synaptophysin	4		1	5
Chromogranin A	3		4	7
CEA			3	3
Desmin	10	1	2	13
S-100	1	2	3	6
EMA	4	1	4	9
SMA	1	1	4	6
AE1:AE3	7	1	2	10
CD99	4			4

*NSE* neuron-specific enolase; *CEA* carcinoembryonic antigen; *EMA* epithelial membrane antigen; *SMA* smooth muscle antigen

Information about the employed treatments and the prognosis was available in 14 cases (Table 3). Chemotherapy alone or in combination with other therapeutical options was used in twelve cases. The most frequent multimodality approach was the combination of chemotherapy with surgery (six cases); radiation therapy was employed in four cases. The mean follow up time was 23 (range 5–76) months. Globally, eight patients died because of the disease, four were alive with disease, and two were free of disease at the respective follow up time (Table 3). Only two patients (13.3%) survived more than 3 years; one of them had a multidisciplinary treatment with surgery, chemotherapy, and radiation, while the other one received chemotherapy and radiotherapy only.

**Table 3:** Treatment options employed and follow up results in the cases under investigation

Article	Treatment	Follow up (months)	Patient status
Bian et al. [6]	Surgery, Chemotherapy.	NA	Dead at 2^nd^ year
Choi et al. [7]	NA	NA	NA
Parkash et al. [8]	Surgery, chemotherapy in both cases	Case 1: 18 Case 2: 15	Case 1: Alive with stable disease. Case 2: Dead.
Venkateswaran et al.,1997 [9]	Chemotherapy	4	Dead
Sàpi et al. [10]	Chemotherapy.	15	Dead
Ostoros et al. [11]	Surgery, Radiation, Chemotherapy.	76	Dead
Cranja et al., 2005 [12]	Radiation, chemotherapy	8	Dead
Karavitakis et al. [13]	Chemotherapy, Surgery, Radiation, Chemotherapy.	34	Free of disease
Quarssani et al., 2011 [14]	Radiation, chemotherapy	53	Alive with disease
Benbrahim et al., 2012 [15]	Chemotherapy	5	Alive with disease
Jian et al. [16]	Chemotherapy	22	Dead.
Cao et al. [17]	Surgery	32	Free of disease
Won et al., 2015 [18]	Chemotherapy	16	Dead
Ikeue et al. [19]	Chemotherapy, surgery, radiation	6	Alive with disease

## Discussion

DSRCT of the pleura are extremely rare; our research detected fifteen cases in the English-language literature, excluding thoracic cases of a different origin. Among 66 DSRCTs managed at the Memorial Sloan-Kettering Cancer Center from 1972 to 2003, only one (1.5%) originated from the pleura, whereas no cases were reported in another case series of 41 patients [8, 22, 23]. There are several DSRCT features which suggest its mesothelial origin: it resembles morphologically the small cell variant of epithelial mesotheliomas, shows dot-like immunostaining for desmin (similar to that described in fetal and adult mesothelial cells), and immunostaining for NSE (as described in large part of malignant mesotheliomas). Choi et al. suggested that the primitive submesothelial cells which give rise to mature mesothelial cells during reparative process may represent the cell of DSRCT origin [7]. Other authors advocated the ‘mesothelioblastoma’ origin, hypothesizing that the tumor originates from a progenitor cell with potential for multilineage differentiation, located in the mesothelium or the submesothelial and subserosal mesenchyme [13].

In our series we found that they commonly involve young patients (the mean age was approximately 25), with a slight predilection for males (60%). History of a previous trauma, asbestos exposure and tobacco smoking were occasionally reported, but it is difficult to link them with the pathogenesis of pleural DSRCT. At the contrary, a specific translocation t (11;22) (p13;q12) unique to this neoplasm has been described; it results from the fusion of Ewing’s sarcoma (*EWSR1*) and Wilms’ tumor (*WT1*) genes and it is believed that the derivative chromosome 22 harbors the functional fusion gene, encoding the chimeric protein which has transcriptional activity, stimulating the uncontrollable proliferation and growth of tumor cells [13]. It has been hypothesized that DSRCT cytogenetically has, in a certain way, the result of the genetic alterations of two different tumors, condensed in a single tumor [26]. This could explain some of the divergences in the differentiation patterns of this tumor, considering for example the potential for multidirectional proliferation of the Wilm’s tumor (which can express vimentin, NSE and cytokeratin), the myogenic differentiation often observed in its stromal component, and the inclination of Ewing’s tumor to show a neurogenic differentiation. Furthermore, it could explain the predilection for young individuals.

Pleural DSRCT mainly requires differentiation from malignant mesothelioma and localized fibrous tumor of the pleura. Both these conditions clinically manifest with chest pain, dyspnea, and pleural effusion, which represent also the most common clinical picture in patients with pleural DSRCT. Furthermore, the clinical examination of the patients is commonly unspecific, as it evidences the absence or reduction of breath sounds in the hemithorax involved or, in advanced cases, bilaterally, with or without crackles in the areas corresponding to residual inflated lungs [16, 17]. Other clinical manifestations, like back or neck pain, arm weakness, and chest compression have been reported, but they are highly unspecific and it is extremely difficult to associate them to DSRCT, localized fibrous tumors or malignant pleural mesothelioma.

The typical imaging findings of malignant mesothelioma include unilateral pleural effusion and thickening of the mediastinal pleura, as well as circumferential and nodular pleural thickening with mild enhancement, in addition to interlobar fissure thickening. At the contrary, a smooth, round or oval, homogeneous mass, with intermediate to high attenuation is typical of localized fibrous tumors on unenhanced CT scans. DSCRT of the pleural have been detected as single lesions or diffuse pleural involvement with pleural effusion, reproducing both the patterns described. Cao et al. reported a detailed description of the CT findings in a case resembling a localized fibrous tumor; the tumor appeared as a large (12 cm in maximum diameter), smooth, oval mass, which formed obtuse angles with the pleural surface, with a homogeneous low attenuation on plain examination (28 Hounsfield Units, HU), and a slight-moderate heterogeneous enhancement on contrast-enhanced CT (38 HU). Moreover, compression of the adjacent lung tissues and enlarged mediastinal lymph nodes were often detected. In cases of advanced tumors contrast enhancement is often heterogeneous, showing low attenuation areas that correspond to myxoid alterations, hemorrhage, necrosis or cystic degeneration; these findings and the pleural effusion can be bilateral in such cases [6, 17]. CT scans for the detection of distant metastases have been performed in most of the cases reviewed; nevertheless, distant metastases are rare, but extension along the crura of the diaphragm to the retroperitoneum has been reported [19]. Also, local invasion of osseous anatomical structures (ribs or vertebrae) has been found, generally evaluated also with MRI or Tc-99m MDP bone scans [9, 13].

The rarity and the unspecific clinical and radiological features mentioned make the diagnosis of DSRCT challenging. Fine needle biopsies were suggestive of DSRCT in two cases in this review, and should be taken into consideration prior to more invasive approaches. Bian et al. and Choi et al. described the cytological findings in DSRCTs evaluated by the Papanicolau test [6, 7]. Scattered, tightly cohesive nests and cords of malignant cells mixed with normal mesothelial cells were showed; the neoplastic cells were characterized by small, uniform nuclei with granular hyperchromatic chromatin, irregular membrane, and inconspicuous nucleoli. Their cytoplasm was generally scanty or even undetectable, with indistinguishable boundaries [6, 7].

Generally, a surgical biopsy (open or thoracoscopic) is necessary to establish the final diagnosis of DSRCT. Video assisted thoracoscopic surgery is minimally invasive and has a high diagnostic potential in cases with pleural effusion and thickening, because it allows the visualization of the lesions and the obtainment of multiple pleural biopsies [27]. Additionally, videothoracoscopic surgery can be performed under local anesthesia, as in the case described by Ikeue et al. [19]. The presence of aggregates of poorly differentiated neoplastic cells with small, round to oval hyperchromatic nuclei, and scanty cytoplasm, surrounded by an abundant desmoplastic stroma, along with immunohistochemistry positivity for vimentin, desmin and NSE, and detection of the specific translocation t (11;22) (p13;q12) are the main elements for the pathological diagnosis of DSRCT.

Open surgery can have both diagnostic and therapeutic purposes. In the latter case, it is generally combined to chemotherapy and radiotherapy, in the context of a multidisciplinary approach. The most recent chemotherapy regiment employed was the “P6 protocol”, which consists of cyclophosphamide, doxorubicin, vincristine, etoposide and ifosfamide. Ikeue et al. reported also the use of pazopanib, and they advocate that from the viewpoint of the quality of life, it may represent a good therapeutic option for this aggressive disease [19]. Among the patients who remained free of disease, one had a multidisciplinary treatment with chemotherapy, surgery, and radiation, and the other one surgery alone. In the latter case, the tumor was completely excised, along with the adjacent lung and pleura, but no information about the management of the enlarged mediastinal lymph nodes were available. A multimodality therapy including surgery was used also in the case of the longest survival (76 months), while in another patient who was alive at 4 years from diagnosis only chemotherapy with radiation therapy were performed.

The main limit of the present review is the small number of cases included, depending on the scarcity of well-documented pleural cases in the current literature, and the strict selection criteria adopted. Nevertheless, the retrieved data are highly representative of the current knowledge on this rare and extremely aggressive disease, and demonstrate the limited effectiveness of the treatments currently adopted. Furthermore, well-described cases are necessary for a better comprehension of the pathophysiology and clinical behavior of the disease, along with new molecular evidences and therapeutic strategies.

## Conclusions

Desmoplastic small round cell tumor of the pleura are extremely rare aggressive tumors which may be challenging to diagnose, because of their rarity and unspecific demographic, clinical, and radiological features. An in-depth knowledge of such features is necessary for the optimal management of patients with pleural DSRCT, and reporting of further cases in the scientific literature is desirable in order to better understand the pathophysiological mechanisms and therapeutic options for this aggressive disease.

## Abbreviations

CEA: Carcinoembryonic antigen
CT: Computed tomography
DSRCT: Desmoplastic small round cell tumor
EWSR1: Ewing’s sarcoma 1 gene
HU: Hounsfield units
MRI: Magnetic resonance imaging
NSE: Neuron-specific enolase
SMA: Smooth muscle actin
Tc-99m MDP: Technetium 99 m–methyl diphosphonate
WT1: Wilms’ tumor 1 gene
